# A multimodal dataset for automating language vitality and endangerment assessment in south-south Nigeria

**DOI:** 10.1038/s41597-025-05337-6

**Published:** 2025-07-01

**Authors:** Moses Ekpenyong, Imelda Udoh, Eno-Abasi Urua, Nse Udoh, Ebitare Obikudo, Ogbonna Anyanwu, Ahmadu Shehu, Esther Sylvanus, Richard Bassey, Unyime Saturday, Temitope Fakiyesi, Celestina-Predia Kekai, Ememobong Udoh, Stella Ansa, Emeka Ifesieh, Gladys Ikhimwin, Unyime Udoeyo, Emem Alexander, Emmanuel Okon, Mfon Ekpe, Benjamin Okon Nyong, Moses Darah, Akpobome Diffre-Odiete, Lucky Ejobee, William Aigbedo, Francis Imoudu, Chima Manda, Mee-eebari Kiine, Doris Ugwu, Aniefon Akpan

**Affiliations:** 1 Department of Computer Science, Faculty of Computing, University of Uyo, Uyo, Nigeria; 2 STEM Centre, University of Uyo, Uyo, Nigeria; 3 Centre for Research and Development, University of Uyo, Uyo, Nigeria; 4 Department of Linguistics and Nigerian Languages, University of Uyo, Uyo, Nigeria; 5 Department of Statistics, University of Uyo, Uyo, Nigeria; 6 Department of Linguistics and Communication Studies, University of Port Harcourt, Port Harcourt, Nigeria; 7 Department of Nigerian Languages and Linguistics, Kaduna State University, Kaduna, Nigeria; 8 Department of Geography and Natural Resources Management, University of Uyo, Uyo, Nigeria; 9 School of General Studies, Nigeria Maritime University, Okerenghigho, Nigeria; 10 Department of Linguistics and Nigerian Languages, University of Calabar, Calabar, Nigeria; 11 Department of Languages and Linguistics, Delta State University, Delta State, Nigeria; 12 Department of Linguistics Studies, University of Benin, Benin, Nigeria; 13 Department of Nigerian Languages, Akwa Ibom State College of Education, Afaha Nsit, Nigeria; 14 Department of Nigerian Languages, National Institute for Nigerian Languages, Off Ogbor Hill, Aba, Nigeria; 15 Department of Languages and Linguistics, Arthur Jarvis University, Calabar, Nigeria; 16 Department of Language and Culture Documentation, Southwestern Edoid Multicultural Institute, Ibadan, Nigeria; 17 Department of English and Communication Studies, Ignatius Ajuru University of Education, Obukuru, Nigeria

**Keywords:** Social sciences, Information technology

## Abstract

In this paper, a multimodal dataset was collected between July 2023 and April 2024 through purposive sampling from a field survey of proper households (households with at least one parent and one child) in South-South Geopolitical Zone of Nigeria. The dataset includes 543 validated responses captured in real-time using an online survey developed with Google Forms. The survey instrument synthesised attributes derived from the United Nations, Educational, Scientific and Cultural Organisation (UNESCO) 2003 Language Vitality and Endangerment (LVE) framework, to capture household-specific data from five households per Local Government Area (LGA). The dataset also includes audio recordings of 108 words selected from the *Swadesh wordlist* and a transcription of the gloss, and tone patterns of each word, for proper description of the language’s speech system. The multimodal dataset can support the analysis of LVE patterns, linguistic trends, and complex interactions affecting language sustainability. It is reusable in linguistic, cultural and social science research, providing a robust resource for examining language diversity and preservation.

## Background & Summary/Introduction

Preserving and revitalising endangered languages are critical to sustaining cultural diversity and intergenerational language transmission^1,2^. In recognition of this urgency, the United Nations, Educational, Scientific and Cultural Organisation (UNESCO) Language Vitality and Endangerment (LVE) 2003 framework^3^ emerged as a fundamental instrument for evaluating language vitality and directing preservation endeavours. The UNESCO LVE tool was developed in 2003, by an ad hoc team of linguists drawn from all over the world, at a UNESCO meeting on safeguarding endangered languages. This tool has 9 factors or indicators useful for assessing language vitality and endangerment, each of which is measured by numerical value of 0–5. The factors which are to be taken together in assessing any language are:

- Intergenerational language transmission
- Absolute number of speakers
- Proportion of speakers within the total population
- Shifts in domains of language use
- Response to new domains and media
- Availability of materials for language education and literacy
- Governmental and institutional language attitudes and policies including official status and use
- Community members’ attitudes toward their own language
- Amount and quality of documentation

The UNESCO LVE was used by researchers in surveys of particular languages. El Kirat^4^ used the LVE to analyse Beni Iznassen Amazigh in Morocco, which recommended the use of the framework for refuting or confirming the validity of conclusions in studies. Both Brenzinger^5^ and Grenoble and Whaley^6^ emphasised the use of these factors in assessing the vitality of languages. Lewis^7^ in applying the UNESCOE LVE to many languages had difficulties defining the degree of endangerment. The Australian Institute of Aboriginal and Torres Strait Islander Studies (AIATSIS) used the UNESCO LVE to survey indigenous languages vitality status and resources in Australia, producing the National Indigenous Languages Survey (NILS) report in 2005. Some adjustments were made with regard to factors, such as replacing notional generations (e.g., children, parents, grandparents) with actual age ranges (0–19, 20–39, 40–59, and 60+). Furthermore, a 10th factor known as ‘status of language programmes’ was added. The Institute of Ethnology and Anthropology (IEA) of the Chinese Academy of Social Sciences (CASS), also used the UNESCO LVE in China. IEA added 3 more factors, and used the 12 to classify over 100 Chinese languages. The UNESCO survey for linguistic vitality and diversity was later developed to collect data for the Atlas of the world’s languages in danger of disappearing as well as develop a methodology for an indicator on the status and trends of linguistic diversity and numbers of speakers of indigenous languages. This tool adopted the LVE components and expanded the factors up to 24, with a factor having a reliability index ranging from 0 to 3. Using the UNESCO LVE criteria, Harrison^8^ examined how environmental, cultural, and socioeconomic factors affect language vitality, drawing attention to the rapid disappearance of indigenous languages worldwide. Moseley^9^ applied the UNESCO LVE indicators to map language endangerment globally, categorising languages by their level of endangerment, using factors such as speaker numbers, language use in various domains, and community support. Gordon^10^ integrated UNESCO’s LVE criteria to assess and classify languages by their vitality and endangerment status, assisting the identification of languages at risk and providing a basis for further linguistic research and preservation efforts. While these works assist the assessment of language vitality and inform preservation efforts, they lack granularity in their data collection, as they rely on broad demographic categories without disaggregating the data by age, gender, and active speakers. Because the UNESCO 2003 LVE framework provides a generic assessment of intergenerational transmission, these studies lack capability for identifying specific communication gaps or indicators contributing to these gaps. Furthermore, most studies have shown limited focus on community-level practices, overlooking the attitudes of individual households, thereby ignoring essential information concerning the acceptability and usability of the language in daily life.

Given the growing threat of language extinction in Nigeria—one of the world’s most linguistically diverse countries—there is an urgent need for structured, granular, and reusable datasets that capture both linguistic and social factors influencing language vitality. This study addresses this need by producing a multimodal dataset that not only captures the UNESCO LVE indicators but also extends them through a synthesis of the data attributes. This approach enables the collection of disaggregated household-level responses, spatial data, and audio-lexical features, facilitating more personalised and context-sensitive inputs. To ensure broad demographic representation, proper households (with at least one parent and one child) were purposively selected from five locations per Local Government Area (LGA) across the South-South zone. Stratified sampling was applied across households to capture diverse perspectives from elders, youths, men, women, and children, reflecting the actual dynamics of language use across age and gender. Data were collected through digital surveys and on-site audio recordings using mobile-enabled tools including Google Forms, WavePad, and UTM Geo Map, supporting real-time entry, geotagging, and consistent field-based validation across sites. The integration of multiple data types enables a richer analysis of the dynamics of language use, transmission, and endangerment, supporting both manual and automated assessments. With the evolution of the digital age, the importance of automating LVE indicators for language ecosystem sustenance has increased—helping to address linguistic changes promptly.

The multimodal data are valuable because, they:

- provide a comprehensive understanding of the language dynamics in South-South Nigeria;
- capture not only the inherent characteristics of the language but also actions, interactions, and changes within the linguistic ecosystem, and important for assessing the vitality of the language;
- preserve linguistic diversity, cultural heritage, and can inform policies for sustainable language development, education, and community engagement; and
- enhance the manipulation and comparative discovery of lexical cognates and relationships between languages.

The synthesised data attributes are novel and have not been previously used in any study, marking a significant contribution to the field. Researchers can reuse these data for:

- *Linguistic studies*–to understand changes and patterns of language use in different domains.
- *Policy advocacy*–to provide evidence for promoting informed language policies and strategies based on measurable vitality outcomes and usage patterns.
- *Community development*–to provide a FAIR (Findable, Accessible, Interoperable and Reusable) resource such as a community-driven app, for enhancing language communities understanding of their preferences and communication needs. Hence, promoting inclusivity.
- *Comparative research*–to assist an effectual comparison of language dynamics between Nigerian regions for broader linguistic and cultural insights attainment.

## Methods

### Data collection

**Fig. 1:**
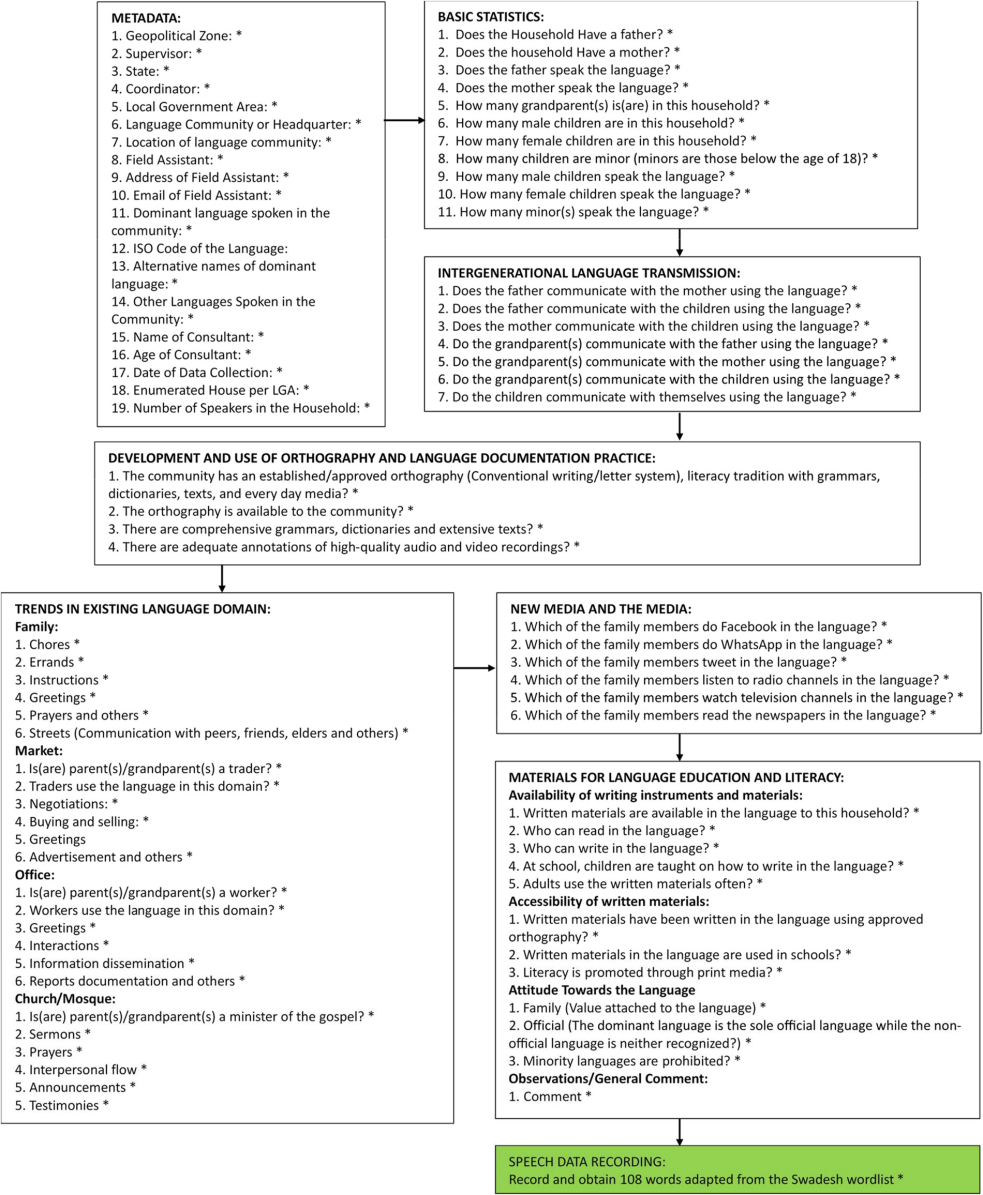
The LEE data collection instrument.

**Fig. 2:**
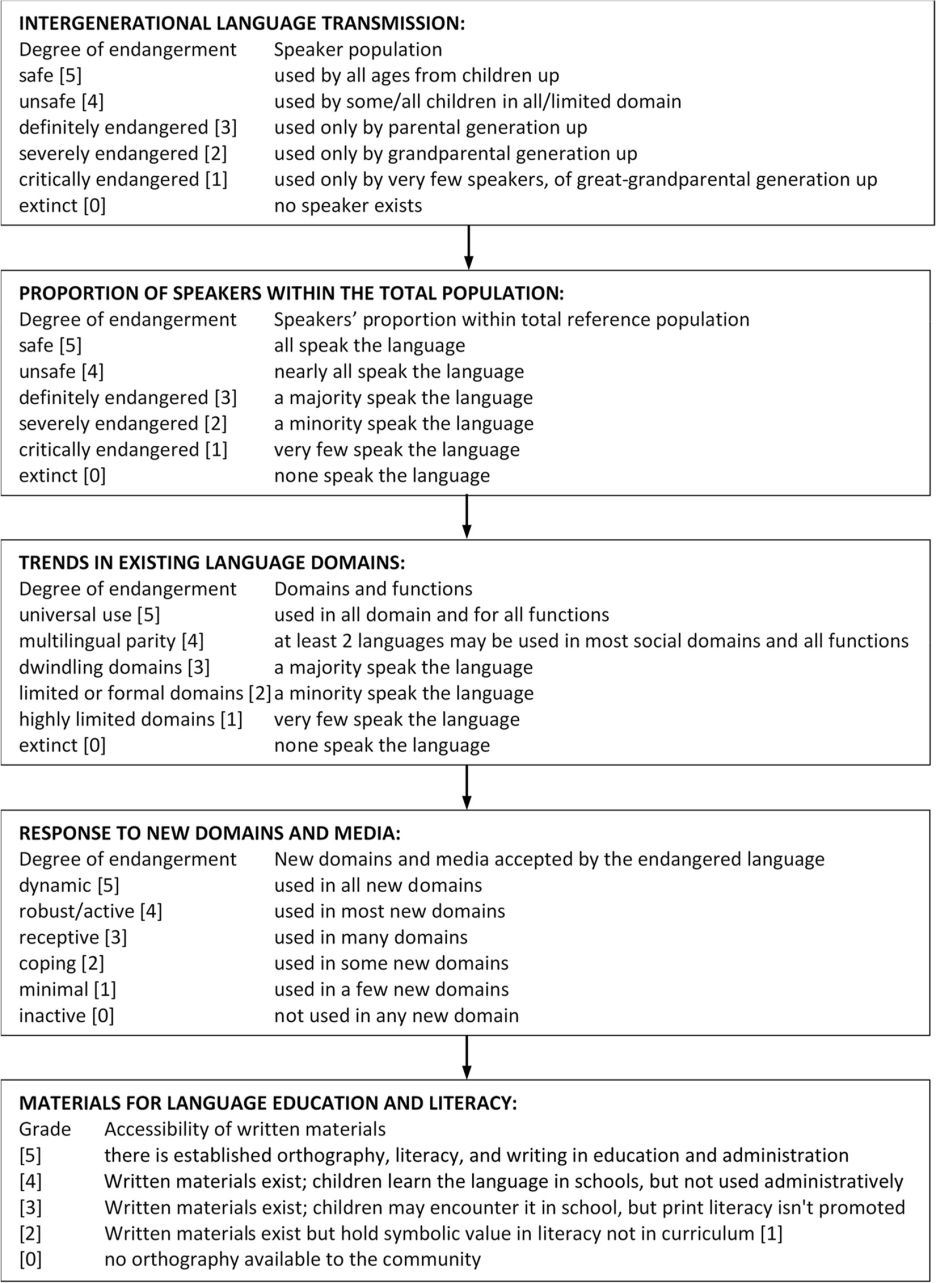
UNESCO 2003 LVE framework.

In a recent National Research Fund (NRF) project, we proposed a novel framework for redefining LVE indicators by integrating both linguistic and spatial/location data^11^. Funded by the Tertiary Education Trust Fund (TETFund), Nigeria, the project synthesised a personalised language ecology and endangerment (LEE) data collection instrument (Fig. 1) from the UNESCO LVE 2003 framework (Fig. 2). Our data collection instrument is an online survey application developed using Google Forms, to enable real-time response capturing and visualisation. Respondents in each household, including elders, youths, men, women, and children, across Southern Nigeria (South-East, South-South and South-West geopolitical zones) participated in the study. The expected product from this research is a *LEE app*, a community-driven application for automating LVE assessment and visualisation across language communities. Furthermore, 108 words selected from the Swadesh Wordlist^12^, our lexicostatistics instrument, were recorded using a competent native speaker or ‘language consultant’, chosen from each community. These recordings assisted the documentation of the language’s speech system (gloss, and tone pattern). While only one representative lexical list is reported per language, each list was carefully designed to reflect standardised lexical coverage and recorded with competent speakers from different LGAs. This approach balances phonological consistency with dialectal representativeness, forming a reliable baseline for tone and speech analysis. Plans for further stratification by age, sex, and proficiency are part of future data collection phases.

**Fig. 3:**
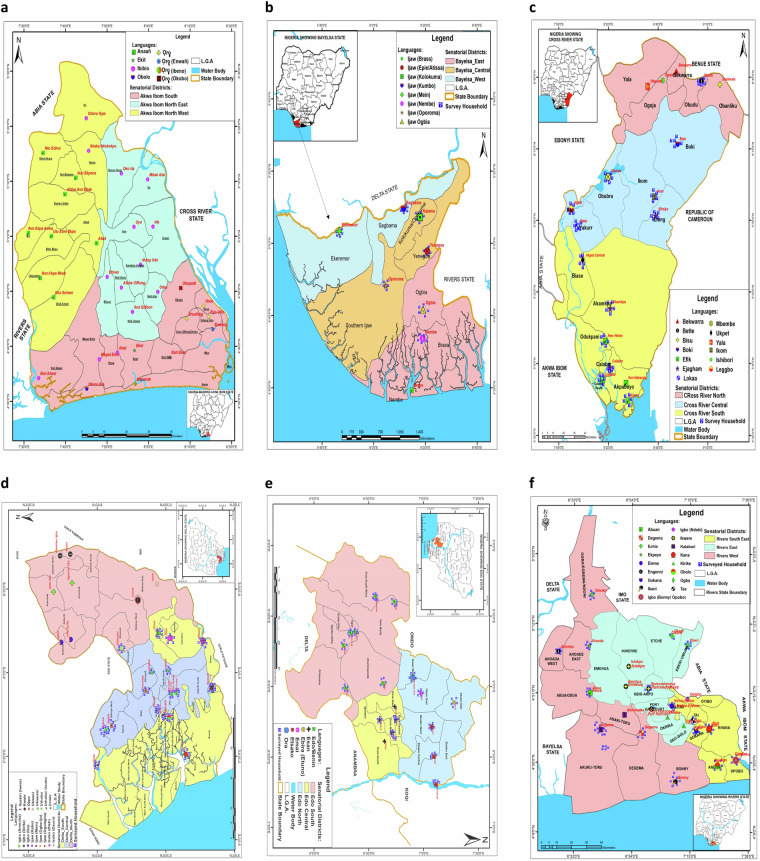
Digitised maps of states in the South-South geopolitical Zone of Nigeria, showing the senatorial districts, LGA, LG headquarters, and randomly distributed households obtained from the NRF project. Validation of the captured locations, including the random distribution of surveyed households were achieved through the use of remote sensing. (**a**) Akwa Ibom State. (**b**) Bayelsa State. (**c**) Cross River State. (**d**) Edo State. (**e**) Delta State. (**f**) Rivers State.

The *UTM Geo Map 3.6.2* was used to ground truth the locations of the respective language communities before actual data capture, facilitating the production of digitised language maps (Fig. 3).

We highlight the various segments of the LEE data collection instrument (Fig. 2) responsible for documenting the LVE assessment criteria, detailing the nature of collected data and novelty in synthesising the UNESCO LVE 2003 indicators (Fig. 2):

- *Meta data:* This segment documents the referenced language community, research assistants, coordinators, supervisors and consultants. This information is important for proper data archiving and referencing.
- *Basic statistics:* This segment enables real-time aggregation of demographic data revealing the specific category of individual households by age, gender and active speakers of the language. This contrasts with the UNESCO 2003 LVE framework which focuses on an overall demographic assessment without proper discrimination by age, gender and active speaker.
- *Intergenerational language transmission*: This segment determines the rate at which the language is transmitted from one generation to another. It specifically identifies the *communication gap* and indicator(s) responsible by aggregating the number of active speakers transmitting the language to the next generation. This contrasts with the UNESCO 2003 LVE framework which merely gives a generic assessment of the household members without identifying the communication gap and indicator(s) contributing to this gap.
- *Development and use of orthography, and language documentation practice*: This segment determines the acceptability and use of the language’s orthography, and the extent of language documentation practices within the community. It aggregates real-time information directly from the individual households who are by extension members of the language community. While the existence of a grammar or orthography is a language-level feature, the goal here was to capture community-level awareness of these resources. This approach provides insight into whether members of the language community are aware of and have access to existing documentation, which is crucial for assessing practical revitalisation potential. We introduced the “not applicable” and “I don’t know” option for questions that don’t apply to the household and for uncertain responses, respectively. This method enables accurate statistical analysis and identification of households or communities in need of sensitisation or intervention.
- *Trend of language use in existing language domain*: This segment evaluates the usage of the language in vital domains and every day functions. A domain represents an activity or item of interaction. We introduce activities peculiar to the family, market, office and religious organisation (e.g., church or mosque), which are missing in the UNESCO 2003 LVE framework. The purpose is to trace the underlying reason(s) or activity pattern(s) influencing the surveyed domain
- *Language acceptance in new domain and media*: This segment evaluates the permissibility of the language in new domain and media. We include the various social media, and relevant questions to derive which household member(s) effectively communicate in the language using these media. This level of detail is absent in the UNESCO 2003 LVE instrument.
- *Materials for language education and literacy:* This segment reveals the active use of the language in education and literacy (availability of writing instruments and materials, accessibility of written materials, and attitude towards the language). This data can be traced back to individual household members, providing more granular insights compared to the UNESCO 2003 LVE framework, which limits the assessment to the community level.

### Data sources

In this study, data were collected from LGAs, within the LG headquarters in the South-South geopolitical zone of Nigeria. The various languages and geographical information of the study area are presented in Supplementary Table 1. Rows highlighted in red indicate locations that could not be accessed as at the time of the study, and reasons for this constraint are given in the Technical Validation section of this paper. Unhighlighted rows represent homogeneous language varieties that were redacted to avoid duplication of wordlists. To digitise the referencing of these locations, we integrated the collected location information (latitude and longitude) with Google Maps and ArcGIS; for enhanced spatial accuracy and visualisation.

### Ethics approval and consent to participate

Ethical clearance for this research was granted by the University of Uyo Health Research Ethics Committee (UNIUYO-HREC) – Reference number: UU/CHS/IHREC/VOL.1/076. All participants provided informed consent after being briefed in a familiar language about the purpose, procedure, and intended use of the study. This include consent for anonymised survey and audio data to be openly shared for research purposes through a data repository.

For participants under the age of 18, informed consent was obtained from a parent or guardian prior to participation. Where necessary, retrospective consent may be documented in accordance with ethical guidelines, particularly in cases where prior interaction between children and their guardians occurred during data collection.

The data were processed and stored in accordance with established best practices for participant confidentiality and ethical research conduct. All datasets were anonymised prior to publication. Personal identifiers—such as names, phone numbers and precise household addresses that could reveal participant-specific locations—were excluded or redacted from the dataset during the pre-processing stage. Audio and text files were therefore labelled using coded metadata corresponding to general locations (e.g., LGA) and language group, without any identifiers linked to specific individuals or households.

### Data characteristics

#### Sociolinguistics Property of the Study Area

The language varieties for this study were selected based on their dominance in LG headquarters. For each LGA, only one dominant language was documented. The distribution of languages by area is as follows^13,14^:

##### *Akwa Ibom State* comprises 5 languages including

Anaañ (spoken in 8 LGAs), Ekịt (spoken in 2 LGAs), Ibibio (spoken in 14 LGAs), Obolo (spoken in 1 LGA) and Ọrọ (spoken in 6 LGAs).

##### *Bayelsa State* comprises 5 languages including

Epie-Attissa (spoken in 1 LGA), Ijaw (spoken in 4 LGAs, Kolokuma (spoken in 1 LGA), Nembe (spoken in 1 LGA) and Ogbia (spoken in 1 LGA)

##### Cross River State comprises 13 languages including

Bekwarra (spoken in 1 LGA), Bette (spoken in 1 LGA), Bisu (spoken in 1 LGA), Bokyi (spoken in 1 LGA), Efik (spoken in 4 LGA), Ejagham (spoken in 3 LGA), Ikom (spoken in 1 LGA), Ishibori (spoken in 1 LGA), Leggbo (spoken in 1 LGA), Lokaa (spoken in 1 LGA), Mbembe (spoken in 1 LGA), Ukpet (spoken in 1 LGA), and Yala (spoken in 1 LGA).

##### *Delta State* comprises 9 languages including

Igbo (spoken in 7 LGAs), Ijaw (spoken in 3 LGAs), Isoko (spoken in 2 LGAs), Kwale (spoken in 1 LGA), Itsekiri (spoken in 1 LGA), Okpe (spoken in 2 LGAs), Ukwani (spoken in 2 LGAs), Urhobo (spoken in 6 LGAs), and Uvwie (spoken in 1 LGA)

##### *Edo state* comprises languages including

Ebira (spoken in 1 LGA), Edo/Benin (spoken in 7 LGAs), Emai (spoken in 1 LGA), Esan (spoken in 5 LGA), Etsako (spoken in 3 LGAs), and Ora (spoken in 1 LGA)

##### *Rivers State* comprises 16 languages including

Abua (spoken in 1 LGA), Degema (spoken in 1 LGA), Echie (spoken in 2 LGAs), Ekpeye (spoken in 1 LGA), Eleme (spoken in 1 LGA), Engenni (spoken in 1 LGA), Gokana (spoken in 1 LGA), Ibani (spoken in 1 LGA), Igbo (spoken in 2 LGAs), Ikwere (spoken in 4 LGAs), Kalabari (spoken in 2 LGAs), Kana (spoken in 1 LGA), Kirike (spoken in 2 LGAs), Obolo (spoken in 1 LGA), Ogbia (spoken in 2 LGAs) and Tee (spoken in 1 LGA).

### Data capturing instruments

#### The Online Survey Instrument

A specification table indicating the questions and available options/inputs of the online survey instrument is presented in Supplementary Table 2. This instrument was divided into two sections, incorporating both the LEE and UNESCO LVE 2003 questionnaires. The format for rendering the options and the number of options required are specified in the second column of Supplementary Table 2.

#### UNESCO LVE 2003 Questionnaire vs. LEE Questionnaire

##### The UNESCO LVE 2003 Questionnaire

This questionnaire manually assesses language vitality using the following criteria: 1) Linguistic vitality and endangerment–measured by the proportion of speakers (father, mother, children and grandparents). 2) Intergenerational language transmission–measured by the rate of language use for all functions/activities in the following domains: family, home, streets, markets and churches. 3) Language use in new domains and media–measured by the rate of language use in new media and related domains, including availability of reading and writing materials, and accessibility to these materials in the community. 4) Attitude towards the language–measured by the level of community awareness (including promotion and protection) of language materials and documentation. To minimise the propagation of errors due to field assistants’ intuition, the UNESCO LVE 2003 questions were automated to systematically collect responses from each households, allowing for the aggregation of responses across households, gender, age, LGA, etc., to produce an overall or general view free from errors.

##### The LEE Questionnaire

This questionnaire is meant to automatically assess language vitality and endangerment using the segments outlined in the Data Collection section. Each LEE question is tailored or personalised to individual households (Supplementary Table 2). Personalising the questions allows the seamless aggregation of responses, distinguishing between different LVE questions based on age, gender, identifying the household member(s), and the very domain functions/activities responsible. This approach facilitates informed policy formulation, decision-making and exposes the root cause of language shift or endangerment. To measure household members’ awareness about language development, we introduced a “not applicable” and an “I don’t know” option for questions that do not apply to the household and for uncertain responses, respectively.

### Survey data acquisition protocol

Protocols were developed for acquiring the multimodal (vitality assessment and lexicostatistics) data. On the vitality assessment data, we developed two protocols, one to capture the survey data and the other to capture the location data. A third protocol was developed to capture the lexicostatistics data. Each protocol is outlined as follows:

#### Protocol 1: Vitality Assessment Data Capture

The steps for obtaining the vitality assessment data include:

- Field assistant visits the LGA Headquarter.
- Identify a native speaker hereinafter referred to as consultant from the Headquarter secretariat/community. Complete the required formalities and access rights were applicable.
- Use a purposive sampling method to select a proper house/household. Select the households from the community that speak the dominant language in the LGA. A total of 5 houses should be selected from each LGA.
- Use stratified random sampling to assess eligibility from a range of recipients within the houses–elders, youths, men, women, and children.
- Administer the LEE survey to the 5 households. May fill into a note and upload later, if the network is not available or is poor.
- Fill in the data for each household into the Google form at: https://forms.gle/8BGD3oFQPDiLTKxu5
- Submit the form.

#### Protocol 2: Location Data Capture

The steps for obtaining the location coordinates (latitude, longitude) of the study area include:

- Launch the UTM Geo Map app from a smart device, when in the vicinity or location of the language community. If the app is not installed, install it from the Google Play Store.
- Select Map Coordinates.
- Select ‘Go to GPS Location’. This step gives the real-time location of the mobile device with GPS accuracy in meters appearing on the screen. Ensure that the GPS accuracy is within an acceptable range. For this study, a GPS accuracy range of 1 to 7.00 metres (i.e., how close the device’s calculated position is from the truth, expressed as a radius), is adopted. A lower GPS accuracy measurement defines the precision of the location of the language community. The coordinates capturing was carried out by Field Assistants. Where the GPS accuracy measurement was too high, such a location was recaptured and tuned to the acceptable accuracy range.
- Select ‘Mark’. A request to enter the ‘Point Name’ will pop up. Enter the location name of the language community.
- Select ‘Save’. Each saved point is stored on the mobile device. To transfer the measured data to an external file, there is an Export tool, which supports different file formats such as XML, CSV, GPX, DXF, TXT, GeoJSON. The exported file format used in this study is the CSV format.
- Select ‘Export/Import’, Export to CSV, type in a filename with.csv extension.
- Select ‘Save’ after you’re done with the previous step.

#### Protocol 3: Lexicostatistics Data Capture

The steps for obtaining the lexicostatistics data include:

- Load the WavePad sound editor software from any device such as the smart phone or laptop computer. If the WavePad software is not installed, install the software using the Google Play Store if using a smart device, or download and install from https://www.nch.com.au/wavepad/download-now.html, if using a smart device.
- Click on the record button (•) at the bottom of the page to start recording. For each word on the list, record three tokens of the word leaving a 2 s silence in-between each token.
- To stop recording, click on the Stop button (▪) at the bottom of the page. To playback and listen to the recording, click on the play button (▶).
- To save the recording, click on ‘File’ at the top of the page, followed by ‘Save As…’; a new page will pop up. Input the file name of the recording and the folder where the file is to be stored. Confirm recording settings–the defaults are.wav file format, 44.1 kHz sample rate. Then click on save at the top of the page (highlighted in blue at the top right-hand corner).
- Cross-check the folder on the phone to confirm that the recorded file is present.
- Access your recording from within the voice recording app and email to: nrflsntf@uniuyo.edu.ng.

#### Computational processing

During the fieldwork, the research team monitored the data collection progress in real-time using the Google Forms backend, ensuring minimal errors and verifying each field worker’s completion status. To further enhance data accuracy, the geographical locations of each community were validated through Google Maps. In this study, the Google Maps interface was manually used to validate the location information of each LG headquarters. The following steps were deployed to achieve this:

- *Step 1:* Open Google Maps (https://maps.google.com).
- *Step 2:* For each location in the dataset, enter the address or coordinates (latitude and longitude) into the Google Maps search bar.
- *Step 3:* Verify that the location is accurate based on surrounding landmarks, regional borders, or additional context provided by the dataset.
- *Step 4:* Document or inform the field assistant of any discrepancies and correct them in the dataset.

Supplementary Information File 1 (LEE_data_unprocessed.xlsx) contains the raw data linked to the Google Form, including both trial and actual captures. Because the South-South geopolitical zone was the first zone to be surveyed, continuous adjustments were made to the Google Form, creating missing attributes and duplicate records within the dataset columns. To ensure data integrity, selected members of the research team comprising of linguists and computational scientists, manually validated each record to create a dataset with unique entries. A Python script was developed to automate the re-integration of unique records into the Google Form, as Google Forms do not automatically remove responses when the linked Google Sheet is updated. The Google Apps Script (Supplementary Information File 2) was written in Python 3.13.0 to connect and transfer the pre-processed (updated) data back into the Google Form, following these steps:

- Step 1: Load the Google Sheet and Form
  - ✓ Open the Google Sheet containing the dataset and verify that each column corresponds to a specific field in the Google Form.
  - ✓ Open the Google Form with matching questions.
- Step 2: Access the Google Apps Script
  - ✓ In the Google Sheet, navigate to Extensions > Apps Script to open the editor.
  - ✓ Remove any existing code, then paste the developed Python script.
- Step 3: Customise the Script
  - ✓ Update the script by replacing ‘Sheet1’ with the actual sheet name.
  - ✓ Replace ‘YOUR_FORM_ID’ with the Google Form ID (found in the form URL).
- Step 4: Run the Script
  - ✓ Click the Run button (play icon) in the Apps Script editor
  - ✓ Authorise the script if prompted. This will clear current entries in the Google Form and replace them with data from the Google Sheet.
- Step 5: Automate the Script by setting up a trigger:
  - ✓ In the Apps Script editor, select Triggers (the clock icon).
  - ✓ Add a new trigger for the updateGoogleForm function, setting the event to either time-driven or on form submission, depending on the desired automation.

The outcome of the computational processing produced the processed dataset for South-South Nigerian region.

To streamline data handling, the Google Forms and associated automation scripts can be integrated to aggregate, validate, and visualise households responses in real-time. A query module may be developed to interface the Google Form, supporting precise aggregation and statistical analysis across demographics such as age, gender, and language domain, ensuring consistent scoring based on recorded LVE indicators. For data validation and reintegration, the custom Google Apps Script developed in Python (Supplementary Information File 2) automates the re-integration of unique responses back into the Google Forms, ensuring synchronisation and real-time updates while minimising manual data cleaning efforts. Planned integration with the Google Maps API would automate geographic and other validation efforts for future data points. These automated workflows will enable a robust and scalable LEE app, suitable for assessing and visualising language vitality and endangerment indicators in real time.

## Data Records

The dataset described in this study has been deposited in Mendeley Data^15^. The dataset includes both the vitality assessment and lexicostatistics data captured across the South-South geopolitical zone of Nigeria.

### The survey dataset

The data records including descriptions and formats of each data associated with this study (Supplementary Table 2) have been discussed in the Methods section. These records are primary data collected from individual households (5 per LGA), and represent real-time responses of the field survey, captured within the respective LG headquarters in the South-South Geopolitical Zone of Nigeria. The data, which were directly elicited from household members within the study area consist of two data collection steps. The first step collects the LEE data while the second step collects the UNESSCO LVE data. Our dataset therefore holds two major data described as follows:

1. Vitality assessment data
  *Meta data:* capture data about the referenced language community, research assistants, coordinators, supervisors and consultants.
  *Basic statistics:* capture demographic statistics about proper households.
  *Intergenerational language transmission information:* capture the number of speakers actually transmitting the language to the next generation, grouping them by age and gender.
  *Development and use of orthography, and language documentation practice:* capture the extent to which the orthography is acceptable for use, and the extent of language documentation practices.
  *Trends of language use in existing language domain:* capture the usage of the language in vital domains and every day functions. Activities peculiar to the family, market, office and religious institutions are introduced, to trace the underlying reason or activity pattern influencing the surveyed domain.
  *Language acceptance in new domain and media:* capture the permissibility of the language in new domain and media.
  *Materials for language education and literacy:* capture the active use of the language in education and literacy, including availability of writing instruments and materials, accessibility of written materials, and attitude towards the language.
2. Lexicostatistics data

*Swadesh wordlist audio files:* capture speech (sound) signals of 108 words.

*Swadesh wordlist transcriptions:* capture annotations of speech system (gloss, and tone pattern) of 108 words.

### File structure

**Fig. 4:**
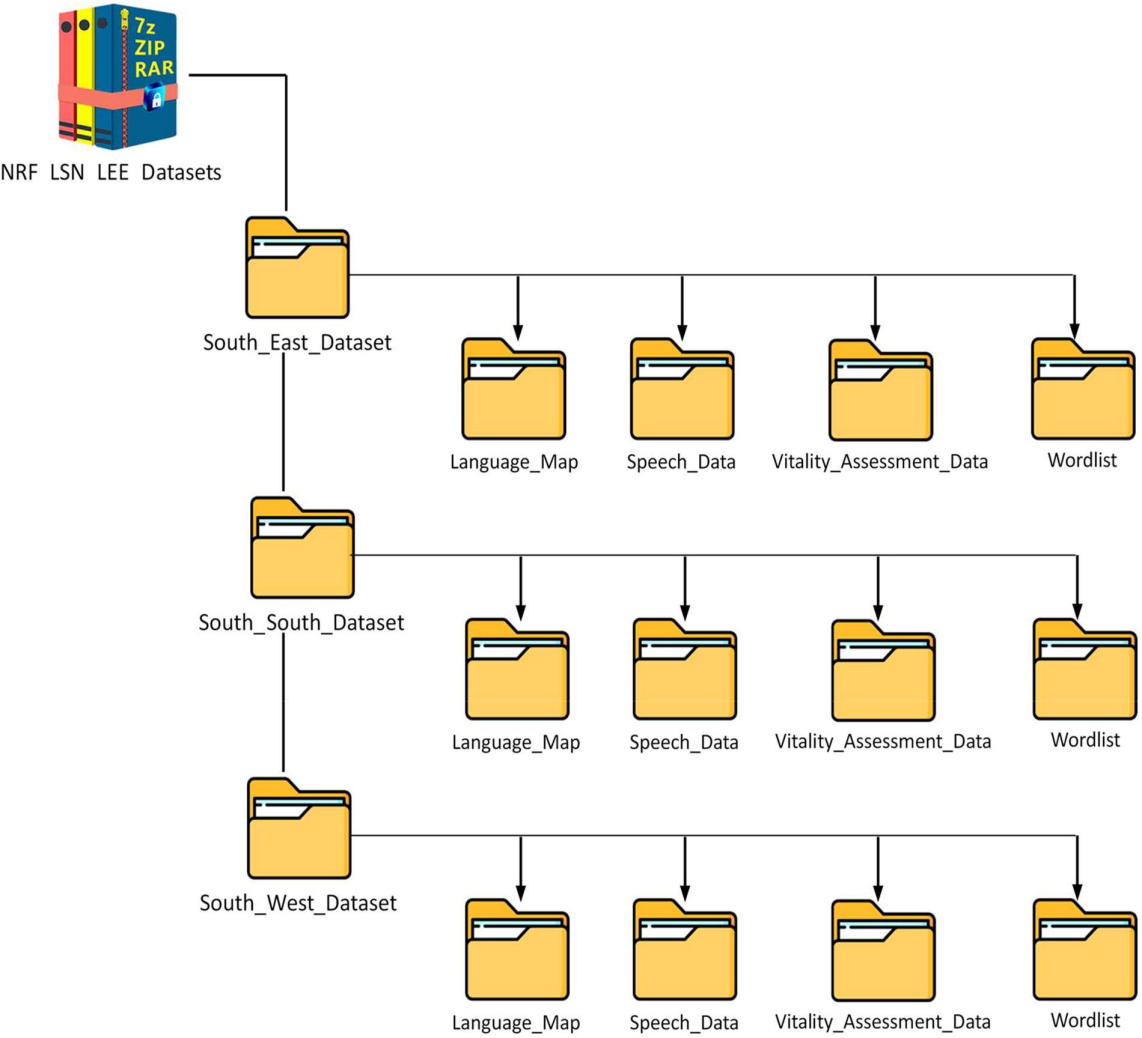
Structure of the LEE dataset.

A file Structure describing the storage folders of this study is presented in Fig. 4. A consistent nomenclature was derived for labelling the different folders. The main folder, ‘NRF_LSN_LEE_Datasets’, describes the type of data; where NRF (National Research Fund) denotes the type of research grant, funded by the Tertiary Education Trust Fund (TETFUND), Nigeria; LSN (languages of Southern Nigeria) describes the group of languages studied; and LEE describes the scope of the research study. The main folder contains three subfolders labelled ‘South-East’, ‘South-South’, and ‘South-West’, each holding four LEE-related data: the language map folder (Language_Map), speech data folder (Speech_Data), vitality assessment data folder (Vitality_Assessment_Data), and the wordlist folder (Wordlist). In this paper, we present only the data for LGAs in the South-South Geopolitical Zone of Nigeria^15^.

### Source data

#### Digitised language map

The collected language data were overlaid onto a geographic map to produce the digitised language map. Developed using the ArcGIS software, the digitised language map combines linguistic information with spatial data to provide insights into the distribution, diversity, and characteristics of languages within the various LGA captured in the study. A verification of the captured locations, including the random distribution of surveyed households were achieved through the use of remote sensing. Digitised language maps of the study area showing the Senatorial Districts, LGA, LGA Headquarters, and randomly distributed households are presented in Fig. 3a-f. Processed language maps can be found at^15^.

#### Speech data

The 108-item Swadesh wordlist was recorded for each language using an Android phone. Since the recordings were made on-site, their quality is inherently suboptimal. Language consultants hired from the respective communities, read the wordlist observing standard audio recording rules. Each recorded audio was then converted to a ‘*.wav’* format, pre-processed to remove all identifying information, and stored in the ‘Speech_Data’ folder, with the filename nomenclature label ‘Geopolitical Zone_State_Senatorial District_LGA_LGA Headquarter_Language’. Processed audio recordings removing consultants’ information can be found at^15^.

#### Vitality assessment data

**Fig. 5:**
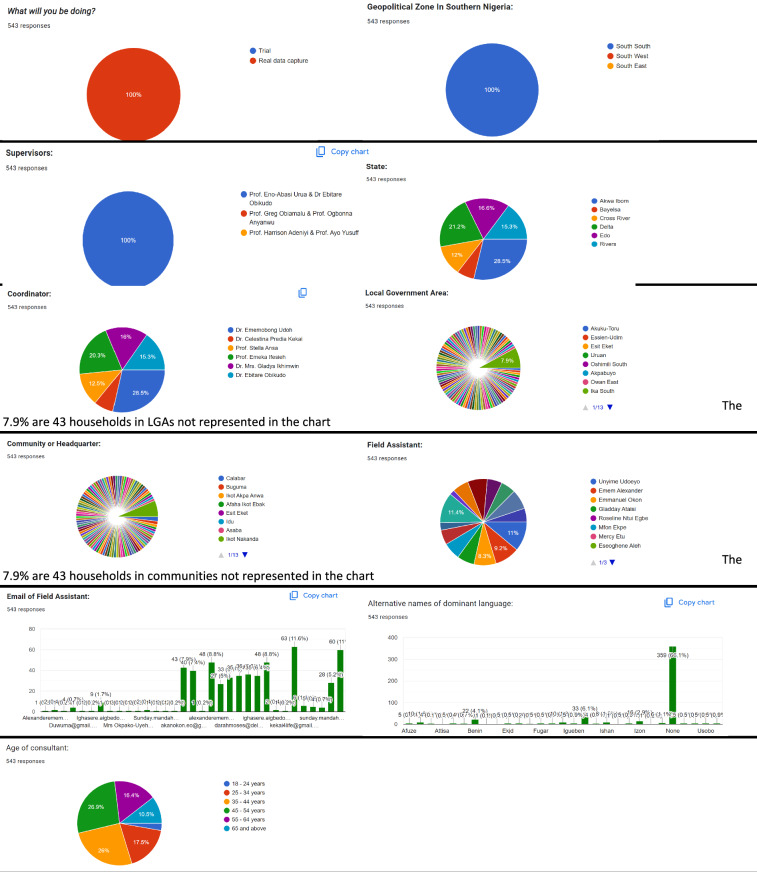
Sample pie charts and bar plots aggregating responses to the LEE metadata questions.

**Fig. 6:**
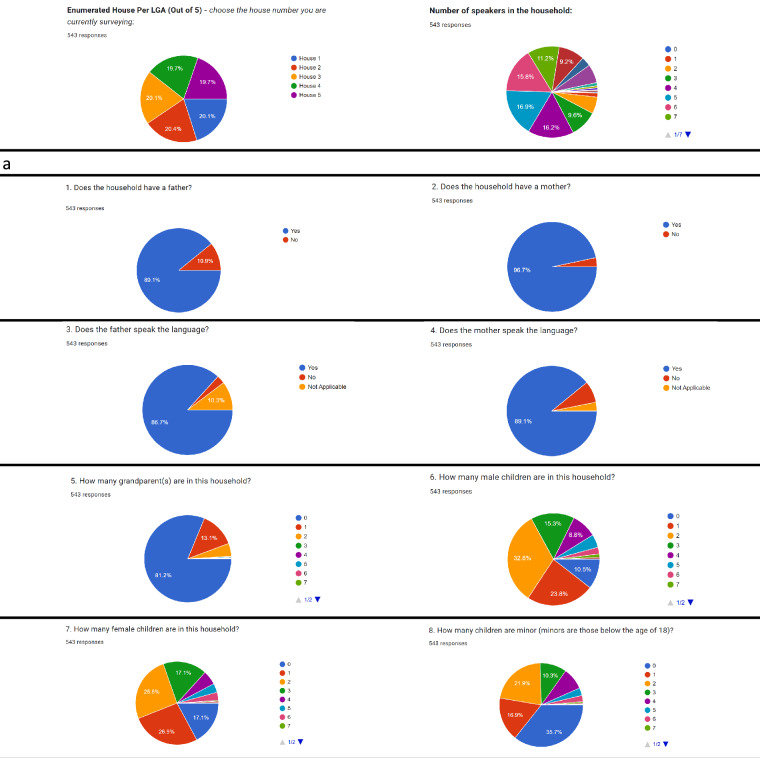
Sample pie charts and bar plots aggregating responses to the first section of LEE questions (section 1). (**a**) Basic statistics.

**Fig. 7:**
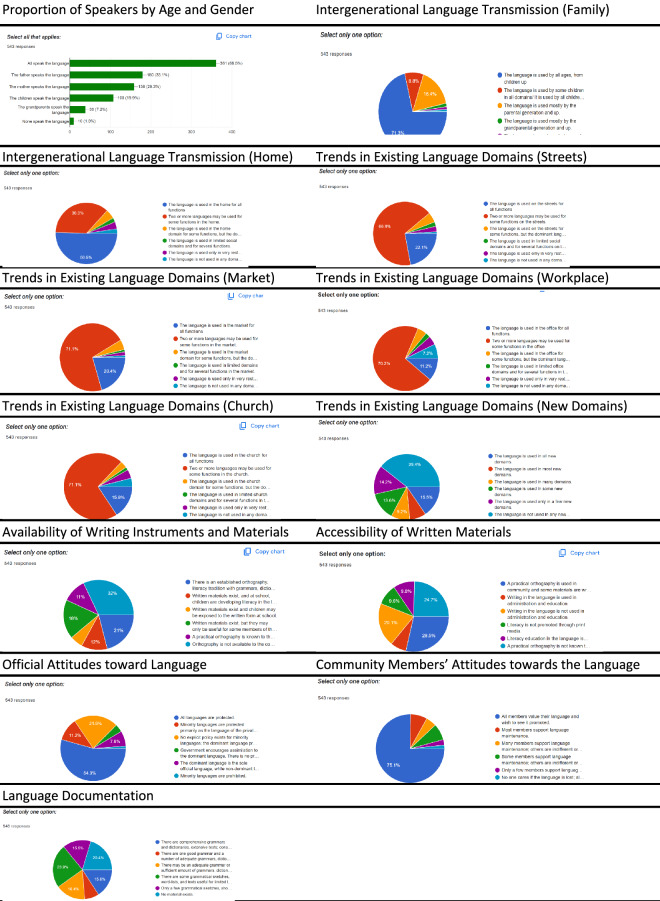
Sample pie charts and bar plots aggregating responses to the UNESCO LVE 2003 questions (section 2).

Sample pie charts and bar plots visualising the survey responses from the field including numerical aggregation of the vitality assessment dataset are presented in Figs. 5–7. For each data category, summary statistics of both the LEE (section 1) and UNESCO LVE 2003 (section 2) assessments are visualised for the two instruments. Processed assessment data can be found at^15^.

#### Transcribed wordlist

The 108-item Swadesh wordlist was transcribed into the language(s) of the respective communities^15^. The transcriptions were done by field assistants, and include the English form, the transcribed form, and the tone pattern. Each transcribed wordlist was later crosschecked by a competent linguist before storage in the ‘Wordlist’ folder, with the filename nomenclature label ‘Geopolitical Zone_State_Senatorial District_LGA_LGA Headquarter_Language’. Processed transcribed wordlists can be found at^15^.

## Technical Validation

### Vitality assessment and lexicostatistics data

The UNESCO LVE 2003 questionnaire has served as a foundational tool for assessing the vitality of local and indigenous languages, but its validation remains largely manual, relying heavily on the intuition of field assistants, field pre-tests, and translations that are time-consuming, subjective, and difficult to replicate across diverse contexts. These limitations, including the absence of spatial data, standardised scoring, and integration with broader datasets, restrict its scalability and utility in dynamic assessments. In contrast, the proposed LEE questionnaire introduces a significant innovation: a spatially-enabled, semi-automated framework that captures georeferenced indicators of language vitality, sociocultural context, and environmental interaction. Our framework is scalable and integrates automated validation checks, supports offline digital data collection, and aligns with open data principles to enhance interoperability, and reproducibility.

In addition to the questionnaire-based assessments, lexicostatistical data were compiled using the 108-item Swadesh wordlist. However, technical validation of this component revealed several challenges that raise concerns about the reliability and validity of the data. As discussed in the next section, these challenges underscore the critical need for expert linguistic oversight, systematic verification, and community-based validation—particularly in under-documented languages where semantic precision and phonological accuracy are essential for meaningful comparative analysis. The proposed LEE app seeks to address these issues by enabling household-level, real-time data input with automated responses and feedback mechanisms. This would reduce reliance on field assistants’ intuition and help minimise data collection errors.

### Expert review and linguistic validation

To ensure the linguistic integrity of the dataset, the compiled wordlists were subjected to validation by a native speaker and expert linguist. This process, which involved a detailed comparison of each word as transcribed by the field assistant with its corresponding audio recording produced by the consultant, enabled the identification and annotation of inconsistencies or errors that may have occurred during fieldwork. Observed issues and their implications, include:

- *Mistranscription:* Field assistants occasionally transcribed lexical items or tonal patterns incorrectly, potentially affecting phonological accuracy and analysis.
- *Incorrect word production:* Consultants at times produced unintended words, introducing variability that may impact lexical consistency.
- *Dialectal variation:* Some lexical items were recorded in dialectal variants, reflecting intra-language diversity that may reduce comparability across speakers.
- *Lexical unfamiliarity:* In certain cases, the consultant or field assistant was unfamiliar with the target word, which may have led to omissions or substitutions.
- *Part-of-speech mismatch:* Consultants sometimes produced a word in the singular form instead of the intended plural, or vice versa, potentially affecting lexical categorisation.
- *Contextual usage:* Some lexical items were produced in sentential or inflected forms rather than as isolated base forms, which may complicate comparative lexical analysis.
- *Substitution with familiar word:* Consultants occasionally replaced target items with semantically related but more commonly used words, potentially influencing the validity of wordlist comparisons.

These issues primarily resulted from the consultants not rehearsing the wordlists beforehand. However, field assistants knowledgeable in the language often guided them. To enhance the utility of the wordlists for further research and analysis, remarks indicating observed issues were added in red font. These annotations flag instances of potential inconsistencies, such as transcription errors, dialectal variation, or part-of-speech mismatches, to support transparency and facilitate data validation. Validated wordlists and audio recordings are labelled with the suffix “…validated,” while those that have not been validated are tagged “…not validated.” A full list of validated and not validated languages are presented in Supplementary Table 2. Rows highlighted in yellow represent languages which wordlist and audio have not been validated by an expert linguist. Rows highlighted in green represent languages which wordlist and audio have been validated by an expert linguist.

### LEE vs. UNESCO LVE 2003 Questionnaire Content

Table 1 compares the contents of the LEE and UNESCO LVE 2003 questions using the following features: purpose, target respondents, focus of responses, data representation, application of results, example domains covered, strengths, weaknesses and error propagation.

**Table 1:** Comparison of questions content between the two instruments.

Feature	Section One: LEE framework	Section Two: UNESCO LVE 2003 framework
Purpose	Synthesises the UNESCO LVE 2003 factors to elucidate the causal LVE factors or agents.	Assesses overall linguistic vitality and endangerment.
Target respondents	Individual households.	Represents views of entire households in each LGA.
Focus of responses	Direct responses from individual households targeted at obtaining specific insights.	Aggregated responses representing the collective perspective of the community.
Data representation	Data reflects the specific, detailed activities or conditions of each household.	Data represents the general consensus or generic condition of the LGA as a whole.
Application of results	Provides detailed, household-level analysis, useful for identifying specific causal LVE agents.	Offers a broader overview of linguistic vitality that can inform regional or national policies.
Example domains covered	Basic statistics, intergenerational language transmission (family members), use of orthography, trends in language use across various domains with detailed activities or causal LVE factors (family, streets, market, office/workplace, religious institution/church), availability of writing instruments, and attitudes toward language use and documentation.	Intergenerational transmission (family members), trends in language use (streets, market, workplace, church), availability of writing instruments, and attitude towards language use and documentation.
Strengths	Allows for more granular, productive and precise interventions targeting specific households and rich content to aggregate broader trends.	Facilitates broader policy-making decisions based on aggregate data.
Weaknesses	May miss broader trends as data is focused on individual households. Broader trends are very possible by deriving or aggregating from individual household categories.	May overlook specific household issues due to its excessive aggregate nature or limited household-specific contents.
Error propagation	Minimal–captures the direct view of each household, including the potentials of automated analyses.	Very high–captures the overall view at each LGA, subject to field assistant’s intuition/judgement.

### LEE vs. UNESCO LVE 2003 Survey Response

Table 2 compares responses obtained during the survey using both instruments. The features compared include: plot types, data focus, granularity, input variables, observed trends, visual clarity, and applications.

**Table 2:** Comparison of obtained responses between the two instruments.

Feature	Section One: LEE data collection instrument	Section Two: UNESCO LVE 2003 framework
Plot type	Pie and bar charts	Pie and bar charts
Data focus	Household-level data, providing detailed distribution across various factors.	Aggregates data at the LGA level, offering a broader overview of linguistic vitality.
Granularity	High granularity with detailed insights into individual household responses.	Lower granularity, focusing on community-wide trends rather than individual variations.
Key variables	See Supplementary Table 2	See Supplementary Table 2
Trends observed	Shows specific trends at the household level, allowing identification and discrimination of outliers and specific patterns.	Displays general trends at the LGA level, highlighting broader demographic patterns.
Visual clarity	May present a complex view due to the higher level of detail, requiring careful, automated interpretation.	Provides more generalised view without detailed content.
Applications	Useful for targeted interventions at the household and can be scaled to broader community contexts.	Better suited for informing high-level, community-wide language policy and planning.

### Limitations

The multimodal dataset available in Mendeley Data^15^, represents the LGAs that were successfully surveyed. Once the proposed LEE app becomes fully operational, it will enable households across Nigeria to participate in the study remotely, potentially expanding the dataset’s coverage and enhancing the scope of future analysis. The primary limitations of this study include:

- *Insecurity Issues:* Specific Senatorial Districts, including Brass LGA in Bayelsa West, Rivers East (Etche, Omuma, Ikwerre, Obio-Okpor, Okrika, Emohua, and Port Harcourt LGAs), and Delta North (Aniocha, Ika, Ndokwa, Oshimili, and Ukwani LGAs), could not be accessed by the field assistants due to the rising cases of piracy and kidnapping concerns.
- *Geographical Inaccessibility:* Challenging topography in Cross River North Senatorial District (Bekwarra, Obanliku, Obudu, Ogoja, and Yala LGAs) limited survey reach.
- *Poor Internet Connectivity:* Across the South-South zone, poor internet connectivity hindered efficient data transmission. Some research assistants had to manually document the LEE questionnaire before uploading. Those with unstable internet service, unable to install WavePad software due to incompatible phone settings, were advised to use their mobile phone recorders. Consequently, all recordings were on-site or field-based and may require enhancements in quality depending on further research purpose.
- *Economic Factors:* Recent inflation and the fuel subsidy crisis led to a 200% increase in transportation costs, straining the project budget and reducing the study’s coverage.

## Supplementary information

Supplementary Information 1

Supplementary Information 2

Dataset 1

## Data Availability

The custom Python code used for automating the re-integration of unique records from the pre-processed dataset into Google Forms is available in Supplementary Information File 2. This code, developed in Python 3.13.0, includes a Google Apps Script for connecting the dataset on Google Sheets with Google Forms. Key parameters, such as sheet names and Google Form IDs, are specified within the code and may require configuration based on the user’s dataset and form structure. Detailed comments are included within the script to guide setup and customisation. Researchers can access the supplementary file along with the manuscript.
