# Supplementary material for: A multimodal dataset for automating language vitality and endangerment assessment in south-south Nigeria
Source: Sci Data. 2025 Jul 1;12:1102. doi: 10.1038/s41597-025-05337-6 (PMC12218391; doi:10.1038/s41597-025-05337-6)
Supplement: Supplementary file 1 — Supplementary Information 1 [file 41597_2025_5337_MOESM1_ESM.doc]

Table 1. Studied languages and their locations. Rows highlighted in yellow represent languages which wordlist and audio have not been validated by an expert linguist. Rows highlighted in green represent languages which wordlist and audio have been validated by an expert linguist. Rows highlighted in red represent locations that could not be accessed by field assistants. Unhighlighted rows represent homogeneous language varieties that were redacted to avoid duplication of wordlists.

| S/N | State | SD | LGN | LGA | LGA Headquarter | Language(same variety group) | Latitude | Longitude |
| --- | --- | --- | --- | --- | --- | --- | --- | --- |
| 1 | AK | NE | 1 | Etinan | Etinan | Ibibio(1) | 4.843265 | 7.844585 |
| 2 | AK | NE | 2 | Ibesikpo Asutan | Nung Udo | Ibibio(1) | 5.014382 | 7.937425 |
| 3 | AK | NE | 3 | Ibiono Ibom | Oko Ita | Ibibio(1) | 5.198181 | 7.893943 |
| 4 | AK | **NE** | 4 | Nsit Atai | Odot | Ibibio(1) | 4.829719 | 8.039068 |
| 5 | AK | NE | 5 | Nsit Ibom | Afaha Offiong | Ibibio(1) | 4.87125 | 7.910442 |
| 6 | AK | NE | 6 | Nsit Ubium | Ikot Edibon | Ibibio(1) | 4.771358 | 7.93701 |
| 7 | AK | NE | 7 | Uruan | Idu | Ibibio(1) | 5.021989 | 8.015581 |
| 8 | AK | NE | 8 | Uyo | Uyo | Ibibio(1) | 5.000877 | 7.950846 |
| 9 | AK | NE | 9 | Itu | Mbak Atai | Ibibio(1) | 5.172781 | 7.999101 |
| 10 | AK | NW | 10 | Abak | Abak | Anaañ(2) | 4.979308 | 7.794434 |
| 11 | AK | NW | 11 | Essien-Udim | Afaha Ikot Ebak | Anaañ(2) | 5.104953 | 7.64898 |
| 12 | AK | NW | 12 | Etim-Ekpo | Utu Etim Ekpo | Anaañ(2) | 4.94877 | 7.588679 |
| 13 | AK | NW | 13 | Ika | Ikot Akpa Anwa | Anaañ(2) | 5.0237 | 7.544213 |
| 14 | AK | NW | 14 | Ikot Ekpene | Ikot Ekpene | Anaañ(2) | 5.176751 | 7.707566 |
| 15 | AK | NW | 15 | Obot Akara | Nto Edino | Anaañ(2) | 5.254067 | 7.584427 |
| 16 | AK | NW | 16 | Oruk Anam | Ikot Ibritam | Anaañ(2) | 4.811217 | 7.617739 |
| 17 | AK | NW | 17 | Ukanafun | Ikot Akpa Nkuk | Anaañ(2) | 4.875364 | 7.55907 |
| 18 | AK | NW | 18 | Ikono | Ibiaku Ntokokpo | Ibibio(1) | 5.185195 | 7.780951 |
| 19 | AK | NW | 19 | Ini | Odoro Ikpe | Ibibio(1) | 5.353452 | 7.74955 |
| 20 | AK | S | 20 | Eket | Eket | Ekịt(3) | 4.645639 | 7.936825 |
| 21 | AK | S | 21 | Esit Eket | Esit Eket | Ekịt(3) | 4.657471 | 8.059783 |
| 22 | AK | S | 22 | Eastern Obolo | Okoro Ete | Obolo | 4.542506 | 7.745597 |
| 23 | AK | S | 23 | Ikot Abasi | Ikot Abasi | Ibibio(1) | 4.56847 | 7.547159 |
| 24 | AK | S | 24 | Mkpat Enin | Mkpat Enin | Ibibio(1) | 4.733435 | 7.750082 |
| 25 | AK | S | 25 | Onna | Abat | Ibibio(1) | 4.620586 | 7.864061 |
| 26 | AK | S | 26 | Mbo | Enwang | Ọrọ(4) | 4.661901 | 8.252359 |
| 27 | AK | S | 27 | Okobo | Okpodi | Ọrọ(4) | 4.846473 | 8.118929 |
| 28 | AK | S | 28 | Oron | Oron | Ọrọ(4) | 4.805486 | 8.232074 |
| 29 | AK | S | 29 | Udung Uko | Eyo Ofin | Ọrọ(4) | 4.77511 | 8.237772 |
| 30 | AK | S | 30 | Uruefong/Oruko | Uruefong | Ọrọ(4) | 4.75283 | 8.164485 |
| 31 | AK | S | 31 | Ibeno | Mkpanak | Ọrọ (Ibeno)(5) | 4.572068 | 7.978367 |
| 32 | BY | C | 1 | Yenagoa | Yenagoa | Epie/Attissa(6) | 4.9625539 | 6.3459034 |
| 33 | BY | C | 2 | Southern Ijaw | Oporoma | Ijaw (Oporomo)(7) | 4.8064185 | 6.0786991 |
| 34 | BY | C | 3 | Nembe | Nembe | Nembe(8) | 4.6603241 | 6.3576409 |
| 35 | BY | E | 4 | Ekeremor | Ekeremor | Ijaw (Mein)(9) | 5.0554734 | 5.77848914 |
| 36 | BY | E | 5 | Kolokuma | Kaiama | Kolokuma(10) | 5.1197489 | 6.2994263 |
| 37 | BY | E | 6 | Ogbia | Ogbia | Ogbia(11) | 4.6619844 | 6.3474724 |
| 38 | BY | W | 7 | Brass | Brass | Ijaw(12) | 4.321793 | 6.246639 |
| 39 | BY | W | 8 | Sagbama | Sagbama | Ijaw (Kumbo)(13) | 5.1586166 | 6.1940635 |
| 40 | CR | C | 1 | Boki | Boje | Bokyi(14) | 6.2760156 | 8.9147077 |
| 41 | CR | C | 2 | Etung | Efreiye | Ejagham(15) | 5.8608314 | 8.7304008 |
| 42 | CR | C | 3 | Ikom | Ikom | Ikọm(16) | 5.970405 | 8.7197682 |
| 43 | CR | C | 4 | Abi | Itigidi | Leggbo(17) | 5.9002133 | 8.02358 |
| 44 | CR | C | 5 | Yakurr | Ugep | Lokəə/aa(18) | 5.7965167 | 8.07399667 |
| 45 | CR | C | 6 | Obubra | Obubra | Mbembe(19) | 6.0850377 | 8.3314753 |
| 46 | CR | N | 7 | Bekwarra | Bekwarra | Bekwarra(20) | 6.701755 | 8.90312 |
| 47 | CR | N | 8 | Obudu | Obudu | Bette(21) | 6.678515 | 9.161285 |
| 48 | CR | N | 9 | Obalinku | Sankwala | Bisu(22) | 6.623674 | 9.269436 |
| 49 | CR | N | 10 | Ogoja | Ogoja | Ishibori(23) | 6.648035 | 8.789773 |
| 50 | CR | N | 11 | Yala | Okpoma | Yala(24) | 6.61156 | 8.662678 |
| 51 | CR | S | 12 | Akpabuyo | Ikot Nakanda | Efịk(25) | 4.8848333 | 8.4815425 |
| 52 | CR | S | 13 | Bakassi | Abana | Efịk(25) | 4.553609 | 8.499605 |
| 53 | CR | S | 14 | Odukpani | New Netim | Efịk(25) | 5.1351377 | 8.3315996 |
| 54 | CR | S | 15 | Calabar South | Calabar | Efịk(25) | 4.91904 | 8.328062 |
| 55 | CR | S | 16 | Akamkpa | Akamkpa | Ejagham(26) | 5.3201949 | 8.3484249 |
| 56 | CR | S | 17 | Calabar Municipality | Calabar | Efịk(25) | 4.9752317 | 8.3421683 |
| 57 | CR | S | 18 | Biase | Akpet-Central | Ukpet(27) | 5.6015602 | 8.1181332 |
| 58 | DE | C | 1 | Okpe | Orerokpe | Okpe(28) | 5.6464433 | 5.89077 |
| 59 | DE | C | 2 | Sapele | Sapele | Okpe(28) | 5.8959383 | 5.682966667 |
| 60 | DE | C | 3 | Ethiope East | Isiokolo | Urhobo(29) | 5.6019465 | 6.0001838 |
| 61 | DE | C | 4 | Ethiope West | Oghara | Urhobo(29) | 5.9361433 | 5.658208333 |
| 62 | DE | C | 5 | Udu | Oto-Udu | Urhobo(29) | 5.4587567 | 5.871323333 |
| 63 | DE | C | 6 | Ughelli North | Ughelli | Urhobo(29) | 5.48124 | 6.011112 |
| 64 | DE | C | 7 | Ughelli South | Oto-Jeremi | Urhobo(29) | 5.425015 | 5.877573 |
| 65 | DE | C | 8 | Uvwie | Effurun | Uvwie(30) | 5.562334 | 5.78011 |
| 66 | DE | N | 9 | Aniocha North | Isele Uku | Igbo (Aniocha)(31) | 6.317656 | 6.484018 |
| 67 | DE | N | 10 | Aniocha South | Ogwashi Uku | Igbo (Aniocha)(31) | 6.182866 | 6.53621 |
| 68 | DE | N | 11 | Oshimili North | Akwukwu Igbo | Igbo (Delta)(32) | 6.196045 | 6.724255 |
| 69 | DE | N | 12 | Oshimili South | Asaba | Igbo (Delta)(32) | 6.196602 | 6.708956 |
| 70 | DE | N | 13 | Ika North East | Owa Oyibo | Igbo (Ika)(33) | 6.190338 | 6.198795 |
| 71 | DE | N | 14 | Ika South | Agbor | Igbo (Ika)(33) | 6.259595 | 6.182132 |
| 72 | DE | N | 15 | Ndokwa West | Utaga-Ogbe | Kwale(34) | 5.703739 | 6.4336182 |
| 73 | DE | N | 16 | Ndokwa East | Aboh | Ukwani(35) | 5.567372 | 6.529846 |
| 74 | DE | N | 17 | Ukwani | Obiaruku | Ukwani(35) | 5.8232051 | 6.1458456 |
| 75 | DE | S | 18 | Patani | Patani | Ijaw (Kabu)(36) | 5.229961 | 6.189886 |
| 76 | DE | S | 19 | Bomadi | Bomadi | Ijaw (Mein)(9) | 5.168735 | 5.912378 |
| 77 | DE | S | 20 | Burutu | Burutu | Ijaw (Ogulagha)(37) | 5.3555009 | 5.5114845 |
| 78 | DE | S | 21 | Isoko South | Oleh | Isoko (Oleh)(38) | 5.470203 | 6.20628 |
| 79 | DE | S | 22 | Isoko North | Ozoro | Isoko (Ozoro)(39) | 5.552805 | 6.236716 |
| 80 | DE | S | 23 | Warri North | Koko | Itsekiri (Iwere)(40) | 5.9988364 | 5.4455002 |
| 81 | DE | S | 24 | Warri South West | Ogbe-Ijo | Ijaw(Ijọ)(41) | 5.4774739 | 5.73293 |
| 82 | DE | S | 25 | Warri South | Warri | Urhobo/Usobo(42) | 5.517108 | 5.74155 |
| 83 | ED | C | 1 | Esan Central | Irrua | Esan(43) | 6.743528 | 6.23559 |
| 84 | ED | C | 2 | Esan North East | Uromi | Esan(43) | 6.711604 | 6.328481 |
| 85 | ED | C | 3 | Esan South East | Ubiaja | Esan(43) | 6.642444 | 6.389443 |
| 86 | ED | C | 4 | Esan West | Ekpoma | Esan(43) | 6.736529 | 6.14288 |
| 87 | ED | C | 5 | Igueben | Igueben | Esan(43) | 6.598029 | 6.237557 |
| 88 | ED | N | 6 | Akoko Edo | Igarra | Ebira (Etuno)(44) | 7.277562 | 6.112518 |
| 89 | ED | N | 7 | Owan East | Afuze | Emai(45) | 7.074735 | 6.285497 |
| 90 | ED | N | 8 | Etsako Central | Fugar | Etsako(46) | 7.093445 | 6.497751 |
| 91 | ED | N | 9 | Etsako East | Agenebode | Etsako(46) | 7.112891 | 6.695408 |
| 92 | ED | N | 10 | Etsako West | Auchi | Etsako(46) | 6.97175 | 6.044816 |
| 93 | ED | N | 11 | Owan West | Sabongida-Ora | Ora(47) | 6.903155 | 5.935484 |
| 94 | ED | S | 12 | Egor | Uselu | Edo/Benin(48) | 6.375091 | 5.612891 |
| 95 | ED | S | 13 | Ikpoba Okha | Idogbo | Edo/Benin(48) | 6.269721 | 5.709371 |
| 96 | ED | S | 14 | Oredo | Benin City | Edo/Benin(48) | 6.333128 | 5.621165 |
| 97 | ED | S | 15 | Orhionmwon | Abudu | Edo/Benin(48) | 6.290085 | 6.011997 |
| 98 | ED | S | 16 | Ovia North East | Okada | Edo/Benin(48) | 6.735346 | 5.399165 |
| 99 | ED | S | 17 | Ovia South West | Iguobazuwa | Edo/Benin(48) | 6.559597 | 5.362799 |
| 100 | ED | S | 18 | Uhunmwonde | Ehor | Edo/Benin(48) | 6.631659 | 5.978668 |
| 101 | RI | E | 1 | Etche | Okehi | Echie(49) | 5.1391567 | 7.1400483 |
| 102 | RI | E | 2 | Omuma | Eberi | Echie(49) | 5.092875 | 7.235174 |
| 103 | RI | E | 3 | Emohua | Emohua | Ikwere(50) | 4.984387 | 6.879752 |
| 104 | RI | E | 4 | Ikwerre | Isiokpo | Ikwere(50) | 4.8730201 | 7.0002631 |
| 105 | RI | E | 5 | Obio-Akpor | Rumuodomaya | Ikwere(50) | 4.752364 | 7.098594 |
| 106 | RI | E | 6 | Port Harcourt | Port Harcourt | Ikwere(50) | 4.7700699 | 7.0173748 |
| 107 | RI | E | 7 | Ogu-Bolo | Ogu | Kirike (51) | 4.88401 | 6.862409 |
| 108 | RI | E | 8 | Okrika | Okrika | Kirike (51) | 4.721294 | 7.202823 |
| 109 | RI | SE | 9 | Eleme | Nchia-Eleme | Eleme(52) | 4.7135501 | 7.2664957 |
| 110 | RI | SE | 10 | Gokana | Kpor | Gokana(53) | 4.7840405 | 7.1394661 |
| 111 | RI | SE | 11 | Oyigbo | Oyigbo | Igbo (Ndoki)(54) | 4.6709427 | 7.36612334 |
| 112 | RI | SE | 12 | Opobo/Nkoro | Opobo | Igbo (Opobo)(55) | 4.8511341 | 7.2271256 |
| 113 | RI | SE | 13 | Khana | Bori | Kana(56) | 4.4841785 | 7.4148538 |
| 114 | RI | SE | 14 | Andoni | Ngo | Obolo(57) | 4.5123889 | 7.5255164 |
| 115 | RI | SE | 15 | Tai | Seakpenwa | Tee(58) | 4.6580334 | 7.2874102 |
| 116 | RI | W | 16 | Abua/Odual | Abua | Abuan(59) | 5.089009 | 6.6407714 |
| 117 | RI | W | 17 | Degema | Degema | Degema(60) | 5.05891 | 6.455892 |
| 118 | RI | W | 18 | Ahoada West | Akinima | Engenni(61) | 4.740472 | 6.855662 |
| 119 | RI | W | 19 | Bonny | Bonny | Ibani(62) | 4.398285 | 7.159058 |
| 120 | RI | W | 20 | Akuku Toru | Abonnema | Kalabari(63) | 4.747797 | 6.766739 |
| 121 | RI | W | 21 | Asari-Toru | Buguma | Kalabari(63) | 5.346201 | 6.657353 |
| 122 | RI | W | 22 | Ahoada East | Ahoada | Ekpeye(64) | 4.852062 | 6.648975 |
| 123 | RI | W | 23 | Ogba/Egbeme/Ndoni | Omoku | Ogba(65) | 4.740485 | 6.855657 |

Key: States: AK=Akwa Ibom, BY=Bayelsa, CR=Cross River, DE=Delta, ED=Edo, RI=Rivers.

Senatorial Districts (SDs): N=North, S=South, E=East, W=West, C=Central, NE=North East, NW=Nort West, SE=South East.

Local Government Number (LGN):
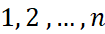
=number of LGAs

Language: x (y)=language spoken (variety), x/y=language/alternative language.

Table 2. The online survey instrument specification table

| Survey question | Option/Input variable | Format | Number of options or values accepted | |
| --- | --- | --- | --- | --- |
| Metadata: | | | | |
| This section collects data about the referenced language community, research assistants, coordinators, supervisors and consultants. | | | | |
| 1. Geopolitical Zone in Southern Nigeria: * | A list of all Geopolitical Zones in Southern Nigeria | List | 1 | |
| 1. Supervisors: * | A list of supervisors | List | 1 | |
| 1. State: * | A list of States in the Geopolitical Zones | List | 1 | |
| 1. Coordinator: * | A list of coordinators | List | 1 | |
| 1. Local Government Area: * | A list of Local Government Areas | List | 1 | |
| 1. Community or Headquarter: * | A list of Local Government Areas Headquarters | List | 1 | |
| 1. Location of language community: * | A text box for accepting the location of the language: Latitude (Lat); Longitude (Long) | Text | 2 | |
| 1. Field Assistant: * | A list of field assistants | List | 1 | |
| 1. Address of Field Assistant: * | A text box for accepting the address of a field assistant | Text | 1 | |
| 1. Email of Field Assistant: * | A text box for accepting the email address of a field assistant | Text | 1 | |
| 1. Dominant language spoken in the community: * | A text box for accepting the dominant language spoken in the community | Text | 1 | |
| 1. ISO Code of the Language: | A text box for accepting the ISO code of the language | Text | 1 | |
| 1. Alternative names of dominant language: * | A text box for accepting the alternatives of the dominant language | Text | 1 | |
| 1. Other Languages Spoken in the Community: * | A text box for accepting other languages spoken in the community | Text | 1 | |
| 1. Name of Consultant: * | A text box for accepting the name of a consultant | Text | 1 | |
| 1. Age of Consultant: * | A list of age ranges:   - 18 - 24 years - 25 - 34 years - 35 - 44 years - 45 - 54 years - 55 - 64 years - 65 and above | Radio button | 1 | |
| 1. Date of Data Collection: * | A calendar for selecting the day, month and year of data collection | Date | 1 | |
| 1. Enumerated House per LGA: * | A list of houses to enumerate (House 1, House 2, …, House 5) | List | 1 | |
| 1. Number of Speakers in the Household: * | A numeric list of speakers (1, 2, 3, …, 50) | Number | 1 | |
| **SECTION ONE (LEE QUESTIONNAIRE)** | | | | |
| Basic Statistics: | | | | |
| This segment collects statistics about proper households. A proper household is that which has at least a parent and a child. | | | | |
| 1. Does the Household Have a father? * | - Yes - No | Radio button | 1 | |
| 1. Does the household Have a mother? * | - Yes - No | Radio Button | 1 | |
| 1. Does the father speak the language? * | - Yes - No - Not Applicable | Radio button | 1 | |
| 1. Does the mother speak the language? * | - Yes - No - Not Applicable | Radio button | 1 | |
| 1. How many grandparent(s) is(are) in this household? * | A numeric list (1, 2, 3, …, 10) | List | 1 | |
| 1. How many male children are in this household? * | A numeric list (1, 2, 3, …, 10) | List | 1 | |
| 1. How many female children are in this household? * | A numeric list (1, 2, 3, …, 10) | List | 1 | |
| 1. How many children are minor (minors are those below the age of 18)? * | A numeric list (1, 2, 3, …, 10) | List | 1 | |
| 1. How many male children speak the language? * | A numeric list (1, 2, 3, …, 10) | List | 1 | |
| 1. How many female children speak the language? * | A numeric list (1, 2, 3, …, 10) | List | 1 | |
| 1. How many minor(s) speak the language? * | A numeric list (1, 2, 3, …, 10) | List | 1 | |
| Intergenerational Language Transmission: | | | | |
| This segment determines the rate at which the language is transmitted from one generation to another. | | | | |
| 1. Does the father communicate with the mother using the language? * | - Yes - No - Not applicable | Radio button | 1 | |
| 1. Does the father communicate with the children using the language? * | - Yes - No - Not applicable | Radio button | 1 | |
| 1. Does the mother communicate with the children using the language? * | - Yes - No - Not applicable | Radio button | 1 | |
| 1. Do the grandparent(s) communicate with the father using the language? * | - Yes - No - Not applicable | Radio button | 1 | |
| 1. Do the grandparent(s) communicate with the mother using the language? * | - Yes - No - Not applicable | Radio button | 1 | |
| 1. Do the grandparent(s) communicate with the children using the language? * | - Yes - No - Not applicable | Radio button | 1 | |
| 1. Do the children communicate with themselves using the language? * | - Yes - No - Not applicable | Radio button | 1 | |
| Development and use of Orthography and Language Documentation: | | | | |
| This segment determines the extent to which the orthography is acceptable for use and documentation of the language. | | | | |
| 1. The community has an established/approved orthography (Conventional writing/letter system), literacy tradition with grammars, dictionaries, texts, and every day media? * | - Yes - No - I don’t know | Radio button | 1 | |
| 1. The orthography is available to the community? * | - Yes - No - I don’t know | Radio button | 1 | |
| 1. There are comprehensive grammars, dictionaries and extensive texts? * | - Yes - No - I don’t know | Radio button | 1 | |
| 1. There are adequate annotations of high-quality audio and video recordings? * | - Yes - No - I don’t know | Radio button | 1 | |
| Trends In Existing Language Domain: | | | | |
| This segment evaluates the usage of the language in vital domains and every day functions. A domain represents an activity or item of interaction. | | | | |
| Family: | | | | |
| 1. Chores * | - All use the language - Parent(s) use the language - Children use the language - None use the language | Radio button | 1 | |
| 1. Errands * | - All use the language - Parent(s) use the language - Children use the language - None use the language | Radio button | 1 | |
| 1. Instructions * | - All use the language - Parent(s) use the language - Children use the language - None use the language | Radio button | 1 | |
| 1. Greetings * | - All use the language - Parent(s) use the language - Children use the language - None use the language | Radio button | 1 | |
| 1. Prayers and others * | - All use the language - Parent(s) use the language - Children use the language - None use the language | Radio button | 1 | |
| 1. Streets (Communication with peers, friends, elders and others) * | - All use the language - Parent(s) use the language - Children use the language - None use the language | Radio button | 1 | |
| Market: | | | | |
| 1. Is(are) parent(s)/grandparent(s) a trader? * | - Yes - No | Radio button | 1 | |
| 1. Traders use the language in this domain? * | - Yes - No | Radio button | 1 | |
| 1. Negotiations: * | - All use the language in this subdomain - Parent(s)/grandparent(s) use the language in this subdomain - Parent(s)/grandparent(s) use another language in this subdomain - Parent(s)/grandparent(s) use the dominant language in this subdomain Children use the language in this subdomain - Children use another language in this subdomain - Children se the dominant language in this subdomain - Language is not used in this subdomain | Check list box | >1 | |
| 1. Buying and selling: * | - All use the language in this subdomain - Parent(s)/grandparent(s) use the language in this subdomain - Parent(s)/grandparent(s) use another language in this subdomain - Parent(s)/grandparent(s) use the dominant language in this subdomain Children use the language in this subdomain - Children use another language in this subdomain - Children use the dominant language in this subdomain - Language is not used in this subdomain | Check list box | >1 | |
| 1. Greetings | - All use the language in this subdomain - Parent(s)/grandparent(s) use the language in this subdomain - Parent(s)/grandparent(s) use another language in this subdomain - Parent(s)/grandparent(s) use the dominant language in this subdomain Children use the language in this subdomain - Children use another language in this subdomain - Children use the dominant language in this subdomain - Language is not used in this subdomain | Check list box | >1 | |
| 1. Advertisement and others * | - All use the language in this subdomain - Parent(s)/grandparent(s) use the language in this subdomain - Parent(s)/grandparent(s) use another language in this subdomain - Parent(s)/grandparent(s) use the dominant language in this subdomain Children use the language in this subdomain - Children use another language in this subdomain - Children use the dominant language in this subdomain - Advertisers use the language in this subdomain - Language is not used in this subdomain | Check list box | >1 | |
| Office: |  |  |  | |
| 1. Is(are) parent(s)/grandparent(s) a worker? * | - Yes - No | Radio button | 1 | |
| 1. Workers use the language in this domain? * | - Yes - No | Radio button | 1 | |
| 1. Greetings * | - All use the language in this subdomain - Parent(s)/grandparent(s) use the language in this subdomain - Parent(s)/grandparent(s) use another language in this subdomain - Parent(s)/grandparent(s) use the dominant language in this subdomain Children use the language in this subdomain - Children use another language in this subdomain - Children use the dominant language in this subdomain - Language is not used in this subdomain | Check list box | >1 | |
| 1. Interactions * | - All use the language in this subdomain - Parent(s)/grandparent(s) use the language in this subdomain - Parent(s)/grandparent(s) use another language in this subdomain - Parent(s)/grandparent(s) use the dominant language in this subdomain Children use the language in this subdomain - Children use another language in this subdomain - Children use the dominant language in this subdomain - Language is not used in this subdomain | Check list box | >1 | |
| 1. Information dissemination * | - All use the language in this subdomain - Parent(s)/grandparent(s) use the language in this subdomain - Parent(s)/grandparent(s) use another language in this subdomain - Parent(s)/grandparent(s) use the dominant language in this subdomain Children use the language in this subdomain - Children use another language in this subdomain - Children use the dominant language in this subdomain - Language is not used in this subdomain | Check list box | >1 | |
| 1. Reports documentation and others * | - All use the language in this subdomain - Parent(s)/grandparent(s) use the language in this subdomain - Parent(s)/grandparent(s) use another language in this subdomain - Parent(s)/grandparent(s) use the dominant language in this subdomain Children use the language in this subdomain - Children use another language in this subdomain - Children use the dominant language in this subdomain - Language is not used in this subdomain | Check list box | >1 | |
| Religious Institution: | | | | |
| 1. Is(are) parent(s)/grandparent(s) a minister of the gospel (preacher/teacher/chorister)? * | - Yes - No | Radio button | 1 | |
| 1. Sermons * | - All use the language in this subdomain - Parent(s)/grandparent(s) use the language in this subdomain - Parent(s)/grandparent(s) use another language in this subdomain - Parent(s)/grandparent(s) use the dominant language in this subdomain Children use the language in this subdomain - Children use another language in this subdomain - Children use the dominant language in this subdomain - Language is not used in this subdomain | Check list box | >1 | |
| 1. Prayers * | - All use the language in this subdomain - Parent(s)/grandparent(s) use the language in this subdomain - Parent(s)/grandparent(s) use another language in this subdomain - Parent(s)/grandparent(s) use the dominant language in this subdomain Children use the language in this subdomain - Children use another language in this subdomain - Children use the dominant language in this subdomain - Language is not used in this subdomain | Check list box | >1 | |
| 1. Interpersonal flow * | - All use the language in this subdomain - Parent(s)/grandparent(s) use the language in this subdomain - Parent(s)/grandparent(s) use another language in this subdomain - Parent(s)/grandparent(s) use the dominant language in this subdomain Children use the language in this subdomain - Children use another language in this subdomain - Children use the dominant language in this subdomain - Language is not used in this subdomain | Check list box | >1 | |
| 1. Announcements * | - All use the language in this subdomain - Parent(s)/grandparent(s) use the language in this subdomain - Parent(s)/grandparent(s) use another language in this subdomain - Parent(s)/grandparent(s) use the dominant language in this subdomain Children use the language in this subdomain - Children use another language in this subdomain - Children use the dominant language in this subdomain - Language is not used in this subdomain | Check list box | >1 | |
| 1. Testimonies * | - All use the language in this subdomain - Parent(s)/grandparent(s) use the language in this subdomain - Parent(s)/grandparent(s) use another language in this subdomain - Parent(s)/grandparent(s) use the dominant language in this subdomain Children use the language in this subdomain - Children use another language in this subdomain - Children use the dominant language in this subdomain - Language is not used in this subdomain | Check list box | >1 | |
| New media and the media: | | | | |
| 1. Which of the family members do Facebook in the language? * | - All - Father - Mother - Grandparent(s) - Children - None | Check list box | >1 | |
| 1. Which of the family members do WhatsApp in the language? * | - All - Father - Mother - Grandparent(s) - Children - None | Check list box | >1 | |
| 1. Which of the family members tweet in the language? * | - All - Father - Mother - Grandparent(s) - Children - None | Check list box | >1 | |
| 1. Which of the family members listen to radio channels in the language? * | - All - Father - Mother - Grandparent(s) - Children - None | Check list box | >1 | |
| 1. Which of the family members watch television channels in the language? * | - All - Father - Mother - Grandparent(s) - Children - None | Check list box | >1 | |
| 1. Which of the family members read the newspapers in the language? * | - All - Father - Mother - Grandparent(s) - Children - None | Check list box | >1 | |
| Materials for language education and literacy: | | | | |
| Availability of writing instruments and materials: | | | | |
| 1. Written materials are available in the language to this household? * | - Yes - No | Radio button | 1 | |
| 1. Who can read in the language? * | - All - Father - Mother - Grandparent(s) - Children - None | Check list box | >1 | |
| 1. Who can write in the language? * | - All - Father - Mother - Grandparent(s) - Children - None | Check list box | >1 | |
| 1. At school, children are taught on how to write in the language? * | - Yes - No - I don’t know | Radio button | 1 | |
| 1. Adults use the written materials often? * | - Yes - No - I don’t know | Radio button | 1 | |
| Accessibility of written materials: | | | | |
| 1. Written materials have been written in the language using approved orthography? | - Yes - No - I don’t know | Radio button | 1 | |
| 1. Written materials in the language are used in schools? * | - Yes - No - I don’t know | Radio button | 1 | |
| 1. Literacy is promoted through print media? * | - Yes - No - I don’t know | Radio button | 1 | |
| Attitude towards the language: | | | | |
| 1. Family: | - All members of this household value their language and wish to see it promoted - Some members of this household support the language - No member supports the use of language and materials in the language | Radio button | 1 | |
| 1. Official (The dominant language is the sole oﬃcial language while the non-oﬃcial language is neither recognized?): | - Yes - No | Radio button | 1 | |
| 1. Minority languages are prohibited? | - Yes - No | Radio button | 1 | |
| **SECTION TWO (UNESCO LVE 2003 QUETIONNAIRE)** | | | | |
| Linguistic Vitality and Endangerment: | | | | |
| 1. Proportion of Speakers by age and gender *: | - All speak the language - The father speaks the language - The mother speaks the language - The children speak the language - The grandparents speak the language - None speak the language | Check list box | >1 | |
| Comment | Enter a comment | Text | 1 | |
| Intergenerational Language Transmission: | | | | |
| 1. Family *: | - The language is used by all ages, from children up - The language is used by some children in all domains/It is used by all children in limited domains - The language is used mostly by the parental generation and up - The language is used mostly by the grandparental generation and up - The language is used mostly by very few speakers, of great-grandparental generation - There exists no speaker. | Radio button | 1 | |
| Comment | Enter a comment | Text | 1 | |
| 1. Home*: | - The language is used in the home for all functions - Two or more languages may be used for some functions in the home - The language is used in the home domain for some functions, but the dominant language penetrates even home domain - The language is used in limited social domains and for several functions - The language is used only in very restricted domains and for a very few functions - The language is not used in any domain and for any function. | Radio button | 1 | |
| Comment | Enter a comment | Text | 1 | |
| Trends in Existing Language Domains; Territory: | | | | |
| 1. Streets*: | - The language is used on the streets for all functions - Two or more languages may be used for some functions on the streets. - The language is used on the streets for some functions, but the dominant language penetrates even the street domain. - The language is used in limited social domains and for several functions on the streets. - The language is used only in very restricted domains and for a very few functions. - The language is not used in any domain and for any function. | Radio button | 1 | |
| Comment | Enter a comment | Text | 1 | |
| 1. Markets*: | - The language is used in the market for all functions - Two or more languages may be used for some functions in the market. - The language is used in the market domain for some functions, but the dominant language penetrates even market domain. - The language is used in limited domains and for several functions in the market. - The language is used only in very restricted domains in the market and for a very few functions. - The language is not used in any domain and for any function. | Radio button | 1 | |
| Comment | Enter a comment | Text | 1 | |
| 1. Work place*: | - The language is used in the oﬃce for all functions. - Two or more languages may be used for some functions in the oﬃce. - The language is used in the oﬃce for some functions, but the dominant language penetrates even oﬃce domain. - The language is used in limited oﬃce domains and for several functions in the oﬃce. - The language is used only in very restricted domains and for a very few functions. - The language is not used in any domain and for any function. | Radio button | 1 | |
| Comment | Enter a comment | Text | 1 | |
| 1. Church*: | - The language is used in the church for all functions - Two or more languages may be used for some functions in the church. - The language is used in the church domain for some functions, but the dominant language penetrates even home domain. - The language is used in limited church domains and for several functions in the church. - The language is used only in very restricted church domains and for a very few functions. - The language is not used in any domain and for any function. | Radio button | 1 | |
| Comment | Enter a comment | Text | 1 | |
| New Domains and Media accepted by the Language: | | | | |
| 1. New domains*: | - The language is used in all new domains. - The language is used in most new domains. The language is used in many domains. - The language is used in some new domains. - The language is used only in a few new domains. The language is not used in any new domains. | Radio button | 1 | |
| 1. Comment | Enter a comment | Text | 1 | |
| 1. Availability of Writing Instruments and Materials*: | - There is an established orthography, literacy tradition with grammars, dictionaries, texts, literature, and everyday media. - Written materials exist, and at school, children are developing literacy in the language. - Written materials exist and children may be exposed to the written form at school. - Written materials exist, but they may only be useful for some members of the community; and for others, they may have a symbolic signiﬁcance. - A practical orthography is known to the community and some material is being written. - Orthography is not available to the community. | Radio button | 1 | |
| Comment | Enter a comment | Text | 1 | |
| 1. Accessibility of Written Materials*: | - A practical orthography is used in community and some materials are written. - Writing in the language is used in administration and education. - Writing in the language is not used in administration and education. Literacy is not promoted through print media. - Literacy education in the language is not a part of the school curriculum. A practical orthography is not known to the community. | Radio button | 1 | |
| Comment | Enter a comment | Text | 1 | |
| Attitude Towards the Language: | | | | |
| 1. Official*: | - All languages are protected. - Minority languages are protected primarily as the language of the private domains. The use of the language is prestigious. - No explicit policy exists for minority languages; the dominant language prevails in the public domain. - Government encourages assimilation to the dominant language. There is no protection for minority languages. - The dominant language is the sole oﬃcial language, while non-dominant languages are neither recognized nor protected. - Minority languages are prohibited. | Radio button | 1 | |
| Comment | - Enter a comment | Text | 1 | |
| 1. Community members*: | - All members value their language and wish to see it promoted. Most members support language maintenance. - Many members support language maintenance; others are indifferent or may even support language loss. - Some members support language maintenance; others are indifferent or may even support language loss. - Only a few members support language maintenance; others are indifferent or may even support language loss. - No one cares if the language is lost; all prefer to use a dominant language. | Radio button | 1 | |
| Comment | - Enter a comment | Text | 1 | |
| 1. Language Documentation*: | - There are comprehensive grammars and dictionaries, extensive texts; constant ﬂow of language materials. Abundant annotated high-quality audio and video recordings exist. - There are one good grammar and a number of adequate grammars, dictionaries, texts, literature, and occasionally updated everyday media; adequate annotated high-quality audio and video recordings. - There may be an adequate grammar or suﬃcient amount of grammars, dictionaries, and texts, but no everyday media; audio and video recordings may exist in varying quality or degree of annotation. - There are some grammatical sketches, word-lists, and texts useful for limited linguistic research but with inadequate coverage. Audio and video recordings may exist in varying quality, with or without any annotation. - Only a few grammatical sketches, short wordlists, and fragmentary texts. - Audio and video recordings do not exist, are of unusable quality, or are completely un-annotated. - No material exists. |  |  | |
| Comment | - Enter a comment | Text | 1 | |
| General Comment*: | | | | |
| Observations and comments: | Enter comments | Text | | 1 |
