# Supplementary material for: A multimodal dataset for automating language vitality and endangerment assessment in south-south Nigeria
Source: Sci Data. 2025 Jul 1;12:1102. doi: 10.1038/s41597-025-05337-6 (PMC12218391; doi:10.1038/s41597-025-05337-6)
Supplement: Supplementary file 2 — Supplementary Information 2 [file 41597_2025_5337_MOESM2_ESM.docx]

// Function to reset the processing state (use only if you need to start over)

function resetProcessingState() {

var scriptProperties = PropertiesService.getScriptProperties();

scriptProperties.deleteProperty('LAST_PROCESSED_ROW');

Logger.log('Processing state has been reset. LAST_PROCESSED_ROW property cleared.');

}

// Function to submit form responses based on spreadsheet data

function submitResponses() {

var formUrl = 'https://docs.google.com/forms/d/1pfDU85B9xTrKHRjs16N99DTJl6WCMr96a7pYQ1KYJOM/edit'; // Ensure this is the correct edit URL of your form

Logger.log('Form URL: ' + formUrl);

try {

var form = FormApp.openByUrl(formUrl); // Open the form by URL

Logger.log('Form opened successfully');

} catch (e) {

Logger.log('Error opening form: ' + e.message);

return;

}

var sheet = SpreadsheetApp.getActiveSpreadsheet().getActiveSheet();

var data = sheet.getDataRange().getValues();

var items = form.getItems();

Logger.log('Starting to process form responses');

// Always start from the second row (index 1 for data array)

var startRow = 1; // Adjusted to ensure we skip the header row

var scriptProperties = PropertiesService.getScriptProperties();

var lastProcessedRow = parseInt(scriptProperties.getProperty('LAST_PROCESSED_ROW')) || startRow;

for (var i = lastProcessedRow; i < data.length; i++) {

// Check if the row has already been processed

if (data[i][data[i].length - 1] === 'Processed') {

Logger.log('Row ' + (i + 1) + ' already processed, skipping.');

continue;

}

Logger.log('Processing row ' + (i + 1)); // Log the actual row number in the sheet (1-based index for readability)

var response = form.createResponse();

var columnIndex = 1; // Start from the second column to skip the timestamp

try {

for (var j = 0; j < items.length; j++) {

var item = items[j];

Logger.log('Item type encountered: ' + item.getType() + ' for item: ' + item.getTitle() + ', ID: ' + item.getId());

// Skip non-form field items

if (item.getType() == FormApp.ItemType.SECTION_HEADER || item.getType() == FormApp.ItemType.PAGE_BREAK) {

Logger.log('Non-form field item skipped: ' + item.getTitle());

continue;

}

var value = data[i][columnIndex]; // Adjust for 0-based index

var itemResponse;

Logger.log('Processing item ' + item.getTitle() + ' with value ' + value);

try {

switch (item.getType()) {

case FormApp.ItemType.TEXT:

itemResponse = item.asTextItem().createResponse(value.toString());

break;

case FormApp.ItemType.PARAGRAPH_TEXT:

itemResponse = item.asParagraphTextItem().createResponse(value.toString());

break;

case FormApp.ItemType.MULTIPLE_CHOICE:

itemResponse = item.asMultipleChoiceItem().createResponse(mapValueToChoice(value, item.asMultipleChoiceItem().getChoices()));

break;

case FormApp.ItemType.CHECKBOX:

var choices = value.split(',').map(function(choice) {

return choice.trim();

});

itemResponse = item.asCheckboxItem().createResponse(choices);

break;

case FormApp.ItemType.LIST:

case FormApp.ItemType.DROPDOWN:

Logger.log('Dropdown choices: ' + item.asListItem().getChoices().map(function(choice) { return choice.getValue(); }).join(', '));

itemResponse = item.asListItem().createResponse(mapValueToChoice(value, item.asListItem().getChoices()));

break;

case FormApp.ItemType.DATE:

itemResponse = item.asDateItem().createResponse(parseDate(value));

break;

case FormApp.ItemType.TIME:

itemResponse = item.asTimeItem().createResponse(parseDate(value));

break;

case FormApp.ItemType.DATETIME:

itemResponse = item.asDateTimeItem().createResponse(parseDate(value));

break;

default:

Logger.log('Unsupported item type encountered: ' + item.getType() + ' for item: ' + item.getTitle() + ', ID: ' + item.getId());

continue;

}

response.withItemResponse(itemResponse);

Logger.log('Item response added: ' + itemResponse);

} catch (e) {

Logger.log('Error processing item ' + item.getTitle() + ': ' + e.message);

}

columnIndex++; // Increment column index after processing each form field

}

response.submit();

Logger.log('Response submitted');

// Mark the row as processed instead of deleting it

sheet.getRange(i + 1, data[i].length + 1).setValue('Processed'); // Ensure the status column is the last column

Logger.log('Marked row ' + (i + 1) + ' as processed.');

// Save the last processed row

scriptProperties.setProperty('LAST_PROCESSED_ROW', i + 1);

} catch (e) {

Logger.log('Error processing row ' + (i + 1) + ': ' + e.message);

// If there's an error, don't mark the row as processed

// The script will retry processing this row on the next run

scriptProperties.setProperty('LAST_PROCESSED_ROW', i);

break;

}

}

// Create a trigger to run this function again after 10 minutes

createTimeDrivenTrigger();

}

// Function to parse date strings in MM/DD/YYYY HH:MM:SS format

function parseDate(dateString) {

if (typeof dateString !== 'string') {

dateString = dateString.toString();

}

var parts = dateString.split(' ');

var dateParts = parts[0].split('/');

var timeParts = parts[1].split(':');

var year = parseInt(dateParts[2], 10);

var month = parseInt(dateParts[0], 10) - 1; // Months are 0-based in JavaScript

var day = parseInt(dateParts[1], 10);

var hour = parseInt(timeParts[0], 10);

var minute = parseInt(timeParts[1], 10);

var second = timeParts.length > 2 ? parseInt(timeParts[2], 10) : 0;

return new Date(year, month, day, hour, minute, second);

}

// Function to map sheet values to form choices

function mapValueToChoice(value, choices) {

var valueStr = value.toString().trim(); // Convert value to string and trim

for (var i = 0; i < choices.length; i++) {

if (choices[i].getValue().trim() === valueStr) {

return choices[i].getValue();

}

}

throw new Error('No matching choice found for value: ' + value);

}

// Function to create a trigger to run the submitResponses function every 10 minutes

function createTimeDrivenTrigger() {

// First, delete all existing triggers to avoid duplicates

deleteAllTriggers();

// Create a new time-based trigger

ScriptApp.newTrigger('submitResponses')

.timeBased()

.everyMinutes(10) // Adjust the interval as needed

.create();

}

// Function to delete all existing triggers

function deleteAllTriggers() {

var allTriggers = ScriptApp.getProjectTriggers();

for (var i = 0; i < allTriggers.length; i++) {

ScriptApp.deleteTrigger(allTriggers[i]);

}

}
